# Characteristics and Functions of *PmHDS*, a Terpenoid Synthesis-Related Gene in *Pinus massoniana* Lamb.

**DOI:** 10.3390/ijms26020457

**Published:** 2025-01-08

**Authors:** Xingyue Ren, Yulu Zhao, Wenya Yu, Jingjing Zhang, Zichen Huang, Mengyang Zhang, Qiong Yu, Kongshu Ji

**Affiliations:** 1 State Key Laboratory of Tree Genetics and Breeding, Nanjing Forestry University, Nanjing 210037, China; 2 Key Open Laboratory of Forest Genetics and Gene Engineering of National Forestry and Grassland Administration, Nanjing 210037, China; 3 Co-Innovation Center for Sustainable Forestry in Southern China, Nanjing Forestry University, Nanjing 210037, China; 4 Beijing National Laboratory for Molecular Sciences, Peking University, Beijing 100871, China

**Keywords:** terpenoids, *Pinus massoniana* Lamb., HDS, abiotic stresses

## Abstract

Terpenoids, abundant and structurally diverse secondary metabolites in plants, especially in conifer species, play crucial roles in the plant defense mechanism and plant growth and development. In *Pinus massoniana*, terpenoids’ biosynthesis relies on both the mevalonate (MVA) pathway and the 2-methyl-D-erythritol-4-phosphate (MEP) pathway, with 1-hydroxy-2-methyl-2-(E)-butenyl-4-diphosphate synthase (HDS) catalyzing the sixth step of the MEP pathway. In this study, we cloned and conducted bioinformatics analysis of the *PmHDS* gene from *P. massoniana*. The results showed that *PmHDS* shares homology with HDS proteins from other species. Analysis of tissue expression patterns indicated that *PmHDS* exhibits the highest expression level in xylem tissue, followed by stems, with significantly lowest expression in the apical meristem. Treatment with NaCl, abscisic acid (ABA), ethylene (ETH), methyl jasmonate (MeJA), and salicylic acid (SA) upregulated the expression of *PmHDS*. Furthermore, we successfully cloned the *PmHDS* promoter (about 2220 bp) and integrated it into a GUS reporter vector, which resulted in GUS activity being observed in various tissues of *Arabidopsis thaliana*. Overexpression of the *PmHDS* gene in *A*. *thaliana* significantly increased the content of carotenoids, chlorophylls a and b, and related enzyme activities, as well as the levels of terpenoid derivatives such as cytokinin (CTK), gibberellic acid (GA), and ABA, thereby enhancing the resistance to those abiotic stresses. These findings suggest that *PmHDS* plays an important role in the terpenoid synthesis pathway. This study provides a theoretical basis for understanding the biosynthesis of terpenoids and lays a foundation for future research on the regulation of terpene synthesis and resistance in molecular breeding.

## 1. Introduction

Terpenoids are a unique category of secondary metabolites synthesized by bacteria, fungi, or plants, primarily fulfilling defensive roles in response to both biotic and abiotic stresses [1]. In plants, these compounds are essential for various physiological and biochemical processes and engage in complex interactions with diverse organisms [2]. They play pivotal roles in deterring herbivores, pests, and pathogenic microorganisms while fostering synergistic relationships with pollinators and beneficial microbes [3,4,5]. All terpenoids are characterized by the presence of five-carbon isoprene (2-methyl-1,3-butadiene, C5H8) as their fundamental structural unit. Depending on the number of isopentenyl diphosphate (IPP) units present in C5H8, terpenoids can be classified into monoterpenes (two units, C10), sesquiterpenes (three units, C15), diterpenes (four units, C20), triterpenes (six units, C30), tetraterpenes (eight units, C40), and polyterpenes (nine or more units exceeding C40) [6]. It has been reported that terpenoids such as the naturally occurring sterols and their derivatives discovered in *Taxus wallichiana* [7], *Artemisia caruifolia* [8], *Panax ginseng* [9], and *Abies fabri* [10] exhibit remarkable pharmacological activities and substantial economic value.

The biosynthetic pathways for terpenoids predominantly consist of two principal routes: the MVA pathway and MEP pathway. Collectively, these pathways produce IPP and its isomer, dimethylallyl diphosphate (DMAPP), which are further converted into direct precursors such as geranyl pyrophosphate (GPP), farnesyl pyrophosphate (FPP), and geranylgeranyl pyrophosphate (GGPP) through enzymatic catalysis. Ultimately, these precursor molecules undergo specific enzyme-mediated reactions to produce various types of terpenoids [11]. Notably, the biosynthesis of the majority of terpenoids relies on the MEP pathway, with HDS serving as the terminal enzyme in this pathway. Belonging to the gcpE protein family, HDS catalyzes the conversion of 2-methyl-D-erythritol-2,4-cyclodiphosphate (MEcPP) into 1-hydroxy-2-methyl-2-(E)-butenyl-4-diphosphate (HMBPP). This reaction is crucial for the subsequent synthesis of IPP and DMAPP, which are subsequently extended through isopentenyl diphosphate transferase-like enzymes and modified by terpenoid synthases to produce a diverse array of terpenoids with various functions.

*HDS* has been extensively studied in various plant species including *Eucommia ulmoides* [12], *Nicotiana tabacum* [13], *Lonicera japonica* [14], *Ginkgo biloba* [15], and *Populus tomentosa* [16]. Research has demonstrated that the expression patterns of the *HDS* gene vary across different plants and tissues. In *Huperzia serrata*, *HDS* expression peaks in leaves while being minimal in roots [17]. Conversely, in *Rauvolfia verticillata*, the highest expression occurs in old leaves, while lower levels are found in stems [18]. Furthermore, *HDS* expression is influenced by abiotic stress. In tea plants, *CsHDS* expression shows significant upregulation after mechanical injury or treatment with MeJA and abscisic acid (ABA) [19]. These findings indicate that HDS may play a role in plant stress responses. Moreover, alterations in *HDS* gene expression have been shown to impact terpenoids’ metabolite levels. Silencing the *LiHDS* gene expression in ‘Siberia’ lilies via utilizing virus-induced gene silencing (VIGS) technology resulted in a significant reduction in the release of aroma-related terpenoids [20]. Conversely, in *Dendrobium officinale*, overexpressing *HDS* enhanced terpenoids’ biosynthesis by accumulating terpene synthase (TPS) [21]. These results suggest that HDS is a key enzyme in the biosynthesis of terpenoids and has potential as a target for modulating terpenoid production in plants. In addition to its role in terpenoid biosynthesis, HDS also plays a crucial role in plant defense. Studies on constitutive subtilisin 3 (*csb3*) mutants in *Arabidopsis thaliana* have revealed that *HDS* could modulate both the MEP pathway and associated resistance factors [22]. These findings highlight the crucial role of HDS in the biosynthesis of terpenoids and its potential as a target in plant breeding and plant protection.

*P. massoniana,* belonging to the *Pinus* subgenus Oleifera Group *(Pinus* sect. *Pinus)* within the genus *Pinus* (Pinaceae), is capable of secreting considerable amounts of terpenoids encompassing monoterpenes, sesquiterpenes, and diterpenes, collectively designated as turpentine. Turpentine has extensive applications in solvents, fragrances, and pharmaceuticals, significantly contributing to its economic value [23,24]. Moreover, it plays a vital role in the defense mechanisms of coniferous trees by releasing secondary metabolites and volatiles from resin ducts in response to damage caused by biotic or abiotic stimuli. These compounds exhibit toxicity towards herbivores and help enhance resistance among conifer species [25,26,27]. The resin-producing capacity of conifers is influenced not only by their genetic quality but also by tree morphology, resin duct structure, needle composition, and environmental factors. The primary sites of terpene component activity are located in the needles and resin ducts [28]. In this study, we focused on the enzymes and regulatory targets within the terpenoids’ molecular metabolic networks, particularly highlighting the pivotal role of HDS in both terpenoids’ synthesis and responses to abiotic stress in *P. massoniana*. Through cloning and analysis of the promoter sequences, we identified that the *PmHDS* promoter is capable of regulating stress-responsive elements and multiple stress factors within the metabolic pathway. Given that an effective genetic transformation platform for *P. massoniana* has yet to be established, we employed heterologous transformation to investigate the function of *HDS* in the MEP pathway during the growth and development of *P. massoniana*. Our findings contribute to a better understanding of the regulation of terpenoids’ biosynthesis pathways in *P. massoniana* and have significant implications for the breeding of high-yielding resin-producing strains of *P. massoniana*.

## 2. Results

### 2.1. Cloning of PmHDS and Bioinformatics Analysis

**Figure 1:**
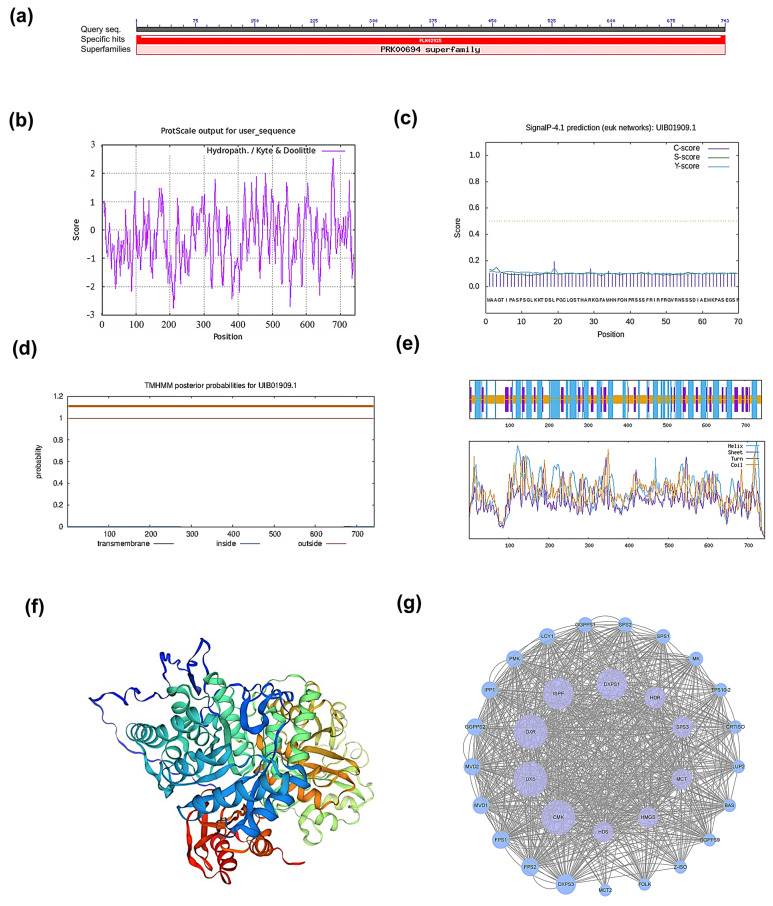
Cloning and sequence analysis of *PmHDS*. (**a**) Analysis of conserved domains in HDS. (**b**) Hydrophilicity profile of the HDS protein. (**c**) Prediction of the signal peptide for HDS. (**d**) Prediction of transmembrane domains in HDS. (**e**) Prediction of secondary structure for the HDS protein. (**f**) Three-dimensional structure prediction of the protein. (**g**) Interaction analysis of HDS proteins.

In this study, we firstly focused on the cloning and bioinformatics analysis of the *PmHDS* gene from *P. massoniana*, with the aim of understanding its function in terpene biosynthesis. We successfully cloned the *PmHDS* gene, which has an open reading frame (ORF) of 2232 base pairs (bps) (Figure S1). This sequence was used for further bioinformatics analysis. Analysis of the conserved structural domain of the HDS protein revealed that the *PmHDS* gene encodes a protein of 743 amino acids and belongs to the superfamily of 4-hydroxy-3-methylbut-2-en-1-yl diphosphate synthase (PRK00694) (Figure 1a). According to physicochemical properties analysis, the theoretical isoelectric point (pI) of the protein is 6.35 and the relative molecular weight of the protein is approximately 82.60 kDa. The protein contains 98 negatively charged residues (aspartic acid, Asp, and glutamic acid, Glu) and 92 positively charged residues (arginine, Arg, and lysine, Lys) (Table S1). The instability coefficient of this protein is 40.38, exceeding the threshold value of 40, indicating that the protein is unstable. The grand average hydropathy index (GRAVY) of the protein is −0.283, suggesting hydrophilic characteristics. The lipid solubility index is 87.66, which further supports the hydrophilic nature of the protein (Figure 1b).

Furthermore, signal peptides and transmembrane region analysis revealed that the protein lacks a signal peptide, confirming its classification as a non-secreted protein (Figure 1c). Predictions regarding transmembrane regions indicate that there are no transmembrane helices present, suggesting that all amino acids spanning positions 1 to 743 reside on or near the membrane surface (Figure 1d). The analysis of the secondary structure of the protein revealed that α-helices constitute 37.82% (281 residues), β-sheets account for 15.88% (118 residues), and disordered coils represent 46.30% (344 residues) (Figure 1e). These findings provide valuable insights into the structural and physicochemical properties of the PmHDS protein.

Moreover, a homologous model template for the tertiary structure of PmHDS was constructed based on a Global Model Quality Estimate (GMQE) value of 0.87 for HDS enzymes. This high GMQE value indicates a reliable model for further structural and functional studies (Figure 1f). Interactions among proteins associated with the MEP and MVA pathways were predicted using *Arabidopsis* as a reference species. Co-expression analyses involving PmHDS and other interacting proteins were performed with *A*. *thaliana* and additional organisms serving as references (Figure 1g).

**Figure 2:**
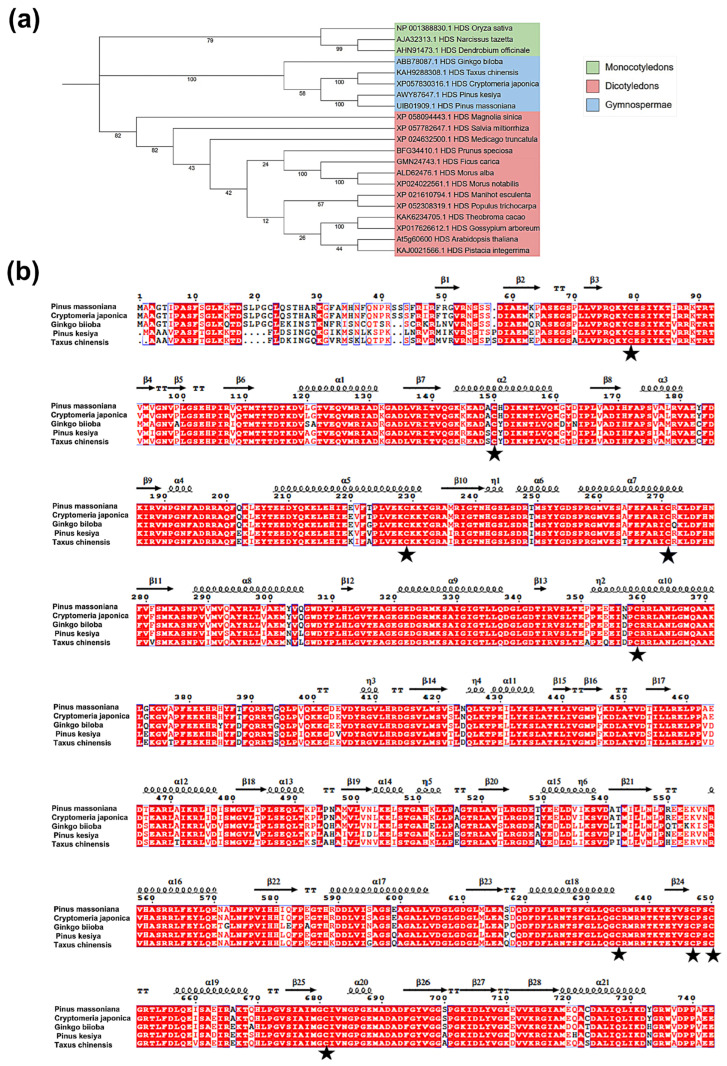
Evolutionary analysis of PmHDS protein. (**a**) Molecular phylogenetic tree of PmHDS. (**b**) Comparative analysis of amino acids in PmHDS from *P. massoniana* and its homologous genes across different species. Note: (**a**) Monocotyledons are represented in light green, Gymnospermae in light blue, and Dicotyledons in red. (**b**) The black pentagonal star indicates the cysteine active site of the HDS enzyme.

To gain insights into the evolutionary relationship of the PmHDS protein within the plant kingdom, we conducted a phylogenetic analysis using MEGA11.0 software. The tree included the PmHDS protein and 21 plant HDS proteins registered in GenBank. Evolutionary distances and clustering were determined using appropriate phylogenetic algorithms. The clustering results indicated that PmHDS grouped with other gymnosperm HDS proteins into a substantial clade, which suggests a shared evolutionary history and potential functional conservation within this group. Specifically, PmHDS exhibited closer relationships with *P. kesiya* within the genus *Pinus* (Figure 2a, Table S2). The close relationship with *P. kesiya* indicates that PmHDS may share similar functional properties and evolutionary constraints with this species. Furthermore, we selected sequences of *P. kesiya* (AWY87647.1), *Cryptomeria japonica* (XP057830316.1), *G. biloba* (ABB78087.1), and *Taxus chinensis* (KAH9288308.1) that exhibited high similarity, up to 90%, to the *P. massoniana HDS* gene to perform multiple sequence alignments using ClustalX2.1 and ESPript 3.0 on their amino acid sequences. Our findings revealed that all plant HDS proteins possess multiple cysteine active sites [4Fe-4S], which may play a role in facilitating iron–sulfur bond formation during substrate reduction [29,30] (Figure 2b). The phylogenetic analysis and multiple sequence alignment provide valuable insights into the evolution and functional conservation of the HDS protein in plants.

### 2.2. Analysis of Tissue-Specific Expression Pattern of PmHDS

**Figure 3:**
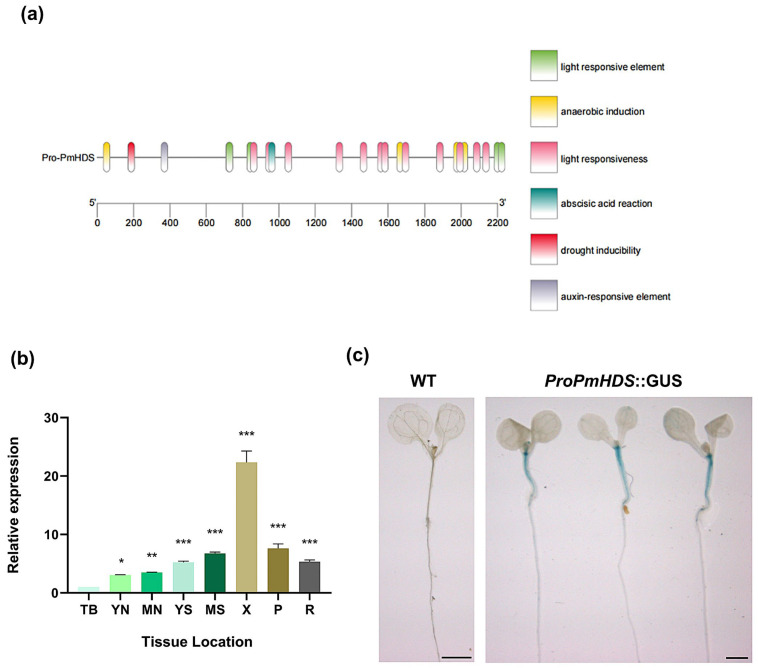
Analysis of tissue-specific expression patterns. (**a**) Analysis of the cis-regulatory elements of the *PmHDS* promoter. (**b**) *PmHDS* expression level in different tissues of *P. massoniana*. (TB: terminal bud; YN: young needle; MN: mature needle; YS: young stem; MS: mature stem; X: xylem; P: phloem; R: root). (**c**) Analysis of pBI121-*proPmHDS*::GUS staining, scale bar = 0.2 mm. The data represent the mean ± SE of three biological replicates. Different numbers of asterisks indicate statistically significant differences (* *p* < 0.05, ** *p* < 0.01, *** *p* < 0.001).

To investigate the tissue-specific expression of the *PmHDS* gene, the 2.2 kb upstream promoter region of the *PmHDS* (Figure S2) gene was cloned, and the cloned promoter sequence was analyzed using PlantCARE. The analysis revealed the presence of core promoter elements (e.g., TATA box and CAAT box), which are essential for transcription initiation. Additionally, the *PmHDS* promoter harbored ABA cis-regulatory elements and drought-responsive elements. (Figure 3a). Based on these predictions, we hypothesized that the expression of the *PmHDS* promoter may be modulated by photoperiods, drought conditions, and plant hormone signaling pathways, particularly those involving ABA.

To assess the expression levels of *PmHDS* across different tissues in *P. massoniana*, qRT-PCR was conducted with *PmTUA* serving as a reference gene. The results indicated that *PmHDS* exhibited peak expression in xylem tissue. Moderate expression levels were observed in both old stems and young stems. Minimal expression was detected in apical buds (Figure 3b). These results suggest a tissue-specific pattern for *PmHDS* within *P. massoniana,* with higher expression in woody tissues such as xylem. To further elucidate the spatial expression profile of this promoter, we successfully constructed a pBI121-*proPmHDS*::GUS fusion expression vector containing the upstream region of *PmHDS* spanning 2.2 kb and stably transformed *A. thaliana*. Histochemical analysis of GUS activity in transformed plants demonstrated that staining driven by the *PmHDS* promoter was predominantly localized to stem tissues, with moderate activity observed in leaves and roots; slight variations were noted among different *A*. *thaliana* accessions regarding their expression patterns (Figure 3c and Figure S3). The tissue-specific expression pattern of *PmHDS*, as revealed by both qRT-PCR and GUS staining, suggests that this gene plays a crucial role in the development and function of woody tissues in *P. massoniana*. The presence of ABA cis-regulatory elements and drought-responsive elements in the *PmHDS* promoter further suggests that the expression of this gene may be modulated by environmental stress factors, such as drought, and plant hormone signaling pathways, particularly those involving ABA.

### 2.3. The Expression Levels of PmHDS in Response to Various Treatments

**Figure 4:**
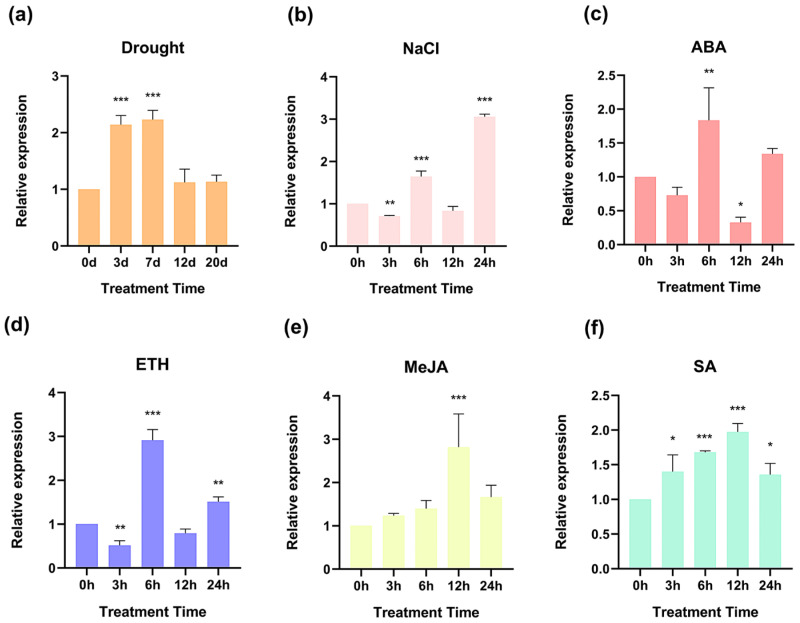
Expression levels under different treatments. (**a**) Expression levels under drought stress. (**b**) Expression levels under NaCl treatment. (**c**) Expression levels under ABA treatment. (**d**) Expression levels under ETH treatment. (**e**) Expression levels under MeJA treatment. (**f**) Expression levels under SA treatment. (* *p* < 0.05, ** *p* < 0.01, *** *p* < 0.001).

To elucidate the expression profile of the *PmHDS* gene under various stress and hormone treatments in *P. massoniana*, we applied 150 mM of NaCl, 100 μM of ABA, 50 μM of ethylene (ETH), 10 mM of MeJA, and 1 mM of SA to healthy two-year-old seedlings. These seedlings were also subjected to drought stress for a specified duration. Across all treatment conditions, *PmHDS* expression significantly increased (Figure 4). After three days and seven days of drought exposure, significant enhancements in *PmHDS* expression were observed at approximately 2.1-fold and 2.2-fold relative to controls, respectively (Figure 4a). Under NaCl-induced stress, overall *PmHDS* expression increased to up to three times that of controls (Figure 4b). In response to ABA, ETH, MeJA, and SA treatments, *PmHDS* exhibited a biphasic expression pattern with peak expressions occurring at specific time points post-treatment (Figure 4c–f). Specifically, peak expressions at six hours post-treatment with ABA and ETH reached approximately 1.8-fold and 2.9-fold greater than controls, respectively (Figure 4c,d). Peak expressions following MeJA and SA treatments occurred at twelve hours post-treatment, with increases of about 2.5-fold and 2.0-fold over control levels, respectively (Figure 4e,f). Overall, the significant increases in *PmHDS* expression across all stress and hormone treatments provide evidence that *PmHDS* is a key gene involved in stress responses in *P. massoniana*, and its expression is modulated by various stress factors and hormones.

### 2.4. Identification, Expression Level Assessment, and Phenotypic Characterization of Transgenic Plants

**Figure 5:**
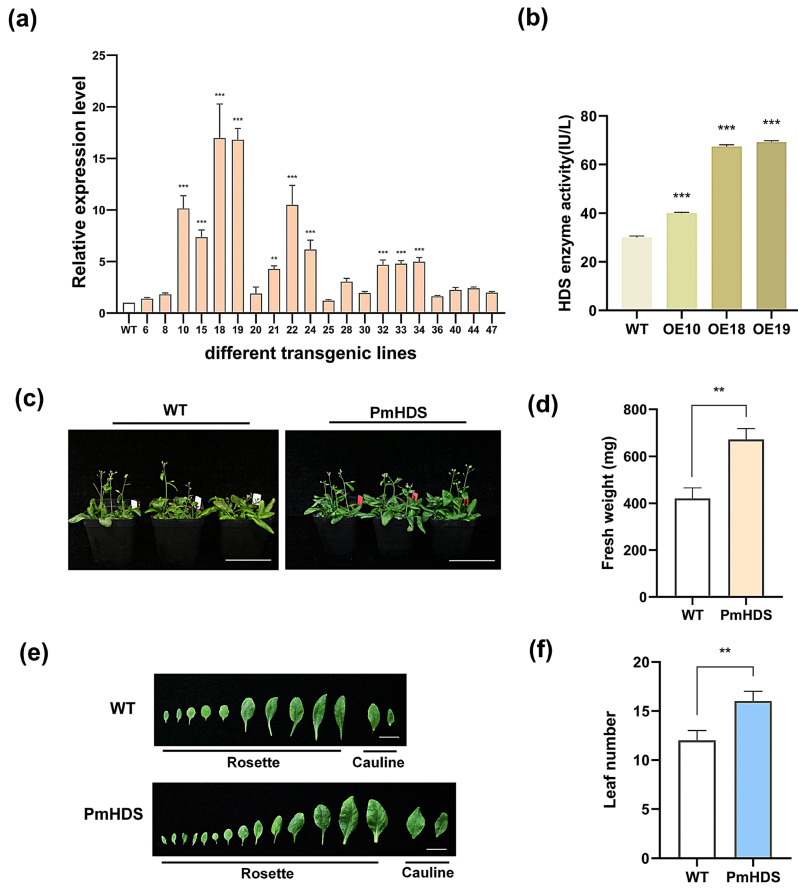
Expression and phenotypic characterization of transgenic *A. thaliana*. (**a**) Relative *PmHDS* expression levels in various overexpression lines. (**b**) Activity of 1-hydroxy-2-methyl-2-(E)-butenyl-4-phosphate reductase (HDS). (**c**) Phenotypic comparison between transgenic plants and wild-type plants, scale bar = 7.0 cm. (**d**) Comparison of fresh weight between transgenic and wild-type plants. (**e**) The quantities of rosette and cauline leaves between transgenic and wild-type plants, scale bar = 1.2 cm. (**f**) Statistical analysis of leaf numbers in transgenic versus wild-type plants. (Different quantities of asterisks indicate significant differences: ** *p* < 0.01, *** *p* < 0.001.)

As no effective genetic transformation platform for *P. massoniana* has been established, we employed heterologous transformation in *A. thaliana* to evaluate the function of *PmHDS* in vivo, especially on plant growth and development. The initial detection of transgenic plants was conducted at the DNA level using gDNA PCR (Figure S4). The results confirmed the presence of the transgene in the transformed plants. Variability in expression levels was observed among 20 different overexpression (OE) lines. Among them, the expression levels of the OE18 and OE19 lines reached the highest levels, approximately 17.0 and 16.8 times that of the control group, respectively. Meanwhile, the expression level of the OE25 line was the lowest, approximately 1.2 times that of the control (Figure 5a). Based on the expression levels, three OE lines (OE10, OE18, and OE19) with elevated expression of the *PmHDS* gene were selected for further experiments. OE18 exhibited the highest expression among the analyzed OE lines. Subsequently, the HDS enzyme activity was measured in transgenic and wild-type *A. thaliana* plants. The results showed that a significant increase in HDS activity was observed in transgenic plants compared to wild-type controls (Figure 5b). Transgenic and wild-type *A. thaliana* plants were cultivated under identical conditions for 30 days. Notably, the leaves of transgenic plants appeared greener than those of their wild-type counterparts (Figure 5c). This phenotypic difference may be attributed to the overexpression of the *PmHDS* gene. Further statistical analysis revealed significant differences in leaf number and fresh weight between transgenic and wild-type plants. Transgenic plants exhibited a greater leaf count per individual plant and an increased fresh weight compared to wild-type plants (Figure 5d–f). The overexpression of the *PmHDS* gene in transgenic *A. thaliana* plants resulted in a significant increase in HDS enzyme activity. This increase was accompanied by phenotypic changes, including greener leaves, increased leaf number, and higher fresh weight compared to wild-type plants. These results suggest that the overexpression of *PmHDS* has positive effects on plant growth and development, potentially through the enhanced biosynthesis of terpene derivatives and related compounds.

### 2.5. Overexpression of PmHDS in Transgenic A. thaliana Enhances Growth Under Hormonal and Abiotic Stress Compared to Wild-Type Plants

**Figure 6:**
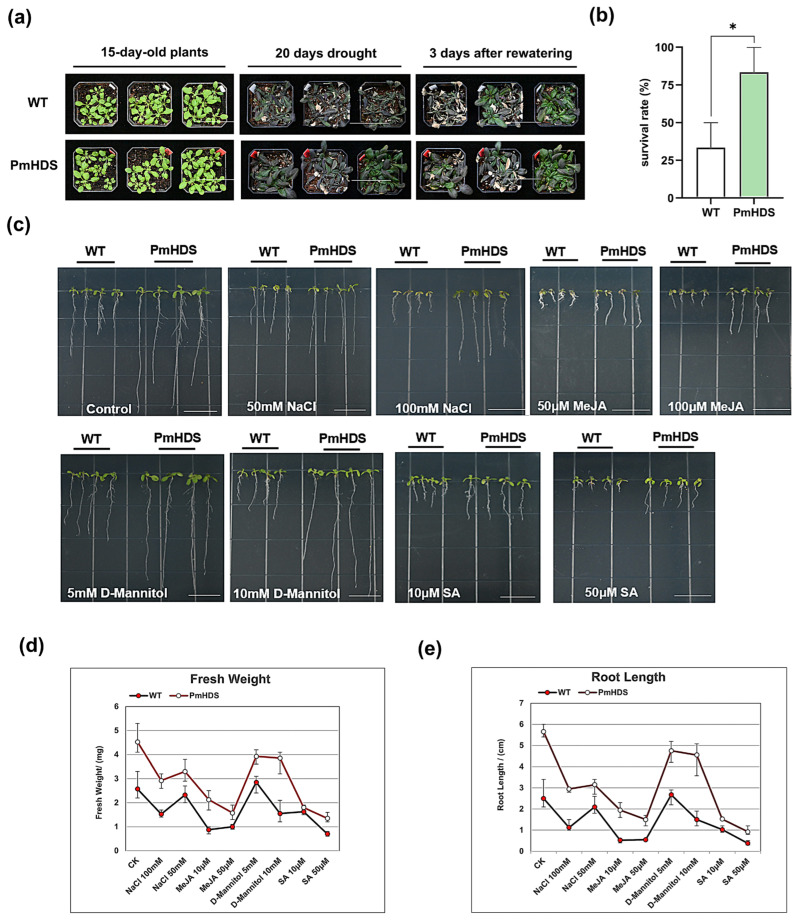
Analysis of the phenotype and physiological indicators of transgenic *A. thaliana* under various treatments. (**a**) Phenotypic observation of *A. thaliana* under drought stress, scale bar = 7.0 cm. (**b**) Statistical analysis of the survival rates of transgenic and wild-type plants following a 3-day rehydration period. (**c**) Phenotypic observation under different treatments, scale bar = 1.5 cm. (**d**) Fresh-weight statistics of transgenic and wild-type plants under different treatments. (**e**) Root length statistics of transgenic and wild-type plants under different treatments. (Note: * indicates a significant difference from the wild-type (WT); * *p* < 0.05.)

To elucidate the function of the *PmHDS*, we selected three transgenic OE lines (OE10, OE18, and OE19) for stress treatment. Initially, both transgenic and wild-type *A. thaliana* plants were subjected to a 20-day drought treatment. Following drought stress, plants were rehydrated for three days, and their growth and survival rates were assessed. Our results indicated that transgenic *A. thaliana* plants exhibited superior growth compared to wild-type plants after 20 days of water deprivation (Figure 6a). After three days of rehydration, transgenic plants had a survival rate of 83.3%, whereas wild-type plants exhibited a survival rate of approximately 33.3% (Figure 6b). To further investigate the function of *PmHDS* in response to stress, transgenic and wild-type *A. thaliana* seeds were sown on 1/2 MS solid culture plates with varying concentrations of NaCl (50 mM and 100 mM), MeJA (10 μM and 50 μM), D-mannitol (5 mM and 10 mM), and SA (10 μM and 50 μM). Phenotypic observations were made after two weeks of growth. Transgenic *A. thaliana* exhibited enhanced resistance to adverse conditions compared to wild-type plants (Figure 6c). Statistical analyses of root length and fresh weight further confirmed that transgenic plants had increased tolerance to stress (Figure 6d,e). The overexpression of the *PmHDS* gene in transgenic *A. thaliana* plants led to enhanced tolerance to drought stress and various hormonal and abiotic stress conditions. This was evident from the superior growth and higher survival rates of transgenic plants compared to wild-type plants under drought stress. Additionally, transgenic plants showed increased resistance to NaCl, D-mannitol, SA, and MeJA treatments, as indicated by their better growth performance, root length, and fresh weight.

### 2.6. Overexpression of PmHDS Enhances Photosynthetic Pigment Content and Increases the Levels of Hormones Associated with Terpenoids’ Synthesis

**Figure 7:**
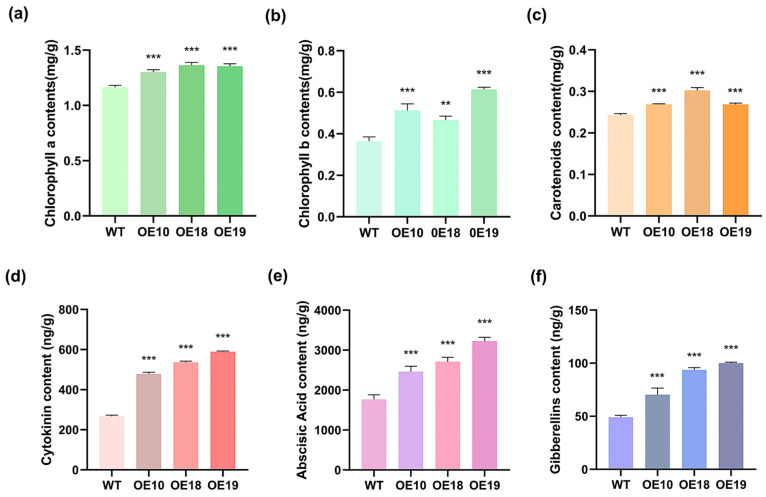
The levels of photosynthetic pigments and the content of related hormones for terpenoid synthesis in transgenic *A. thaliana*. (**a**) Contents of chlorophyll a. (**b**) Contents of chlorophyll b. (**c**) Contents of carotenoids. (**d**) Contents of CTK. (**e**) Contents of ABA. (**f**) Contents of ETH. (Note: ** *p* < 0.01, *** *p* < 0.001.)

The HDS gene plays a pivotal role in the MEP synthesis pathway, which is crucial for the production of terpenoids. As important terpenoids, plant photosynthetic pigments including chlorophyll b, chlorophyll a and carotenoids and hormones such as cytokinin (CTK), abscisic acid (ABA), and gibberellins (GA) significantly regulate plant growth and development. To evaluate the effects of *PmHDS* on terpenoid metabolites, chlorophyll a, chlorophyll b, carotenoids, CTK, ABA, and GA contents were quantified in both wild-type and three transgenic *A. thaliana* lines overexpressing *PmHDS* (OE10, OE18, and OE19). Compared to the wild-type plants, all three overexpression lines showed significantly elevated levels of chlorophyll a, chlorophyll b, and carotenoids (Figure 7a–c). Notably, OE19 displayed the highest chlorophyll b content at 0.61 mg/g, while OE18 exhibited peak levels of chlorophyll a and carotenoids at 1.36 mg/g and 0.30 mg/g, respectively (Figure 7a–c). All three transgenic lines showed significantly increased levels of CTK, ABA, and GA compared to the wild-type plants (Figure 7d–f). Among them, OE19 demonstrated the highest expression levels for hormones: CTK at 588.7 ng/g, ABA at 3223.6 ng/g, and GA at 100.0 ng/g (Figure 7d–f). The overexpression of *PmHDS* in *A. thaliana* led to the enhanced production of photosynthetic pigments, including chlorophyll a, chlorophyll b, and carotenoids. This increase in pigment content suggests that transgenic plants may have improved photosynthetic efficiency and energy capture capabilities. Furthermore, the elevated levels of CTK, ABA, and GA in the transgenic lines indicate that *PmHDS* overexpression also affects the synthesis of hormones associated with terpenoid biosynthesis. These hormones play critical roles in regulating plant growth, development, and stress responses.

### 2.7. Overexpression of PmHDS in A. thaliana Affects the Expression of Genes Related to Terpenoid Synthesis Pathway

**Figure 8:**
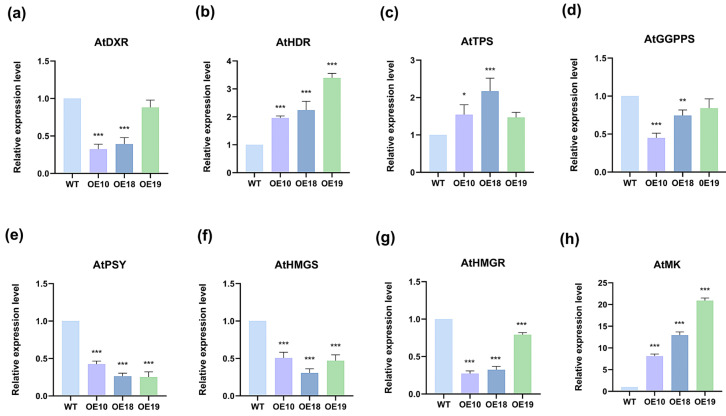
Expression of genes associated with the terpenoids synthesis pathway in *A. thaliana* overexpressing *PmHDS*. (**a**) Relative expression level of *AtDXR* gene. (**b**) Relative expression level of *AtHDR* gene. (**c**) Relative expression level of *AtTPS* gene. (**d**) Relative expression level of *AtGGPPS* gene. (**e**) Relative expression level of *AtPSY* gene. (**f**) Relative expression level of *AtHMGS* gene. (**g**) Relative expression level of *AtHMGR* gene. (**h**) Relative expression level of *AtMK* gene. (Note: * indicates a significant difference from the wild-type (WT); * *p* < 0.05, ** *p* < 0.01, *** *p* < 0.001.)

The biosynthesis of terpenoids in plant cells is a complex and multifaceted process regulated by various enzymes, including 1-Dehydro-D-xylose-5-phosphate synthase (DXS), 1-deoxy-D-xylulose-5-phosphate-reductoisomerase (DXR), HDS, 3-Hydroxy-3-methyglutary-CoA synthase (HMGS), 3-Hydroxy-3-methyglutary-CoA reductase (HMGR), 5-Phosphate-mevalonate kinase (PMK), mevalonate kinase (MK), terpene synthases (TPSs), and geranylgeranyl pyrophosphate synthase (GGPPS). To elucidate the impact of *PmHDS* overexpression on the expression profiles of genes involved in terpenoid synthesis pathways, we selected *AtDXR* and *AtHDR* from the MEP pathway (where *AtDXR* is positioned upstream and *AtHDR* downstream of the *HDS* gene), as well as *AtHMGS*, *AtHMGR*, and *AtMK* from the MVA pathway; *AtGGPPS* from the *GGPPS* pathway; and key terpenoid genes (*AtTPS*). The expression levels of these candidate genes were assessed using qRT-PCR. Wild-type *A. thaliana* was utilized as a negative control group, with *AtActin2* (gene ID: 821411) serving as a reference gene. Our results demonstrated that the expression levels of *AtDXR*, *AtGGPPS*, *AtPSY*, *AtHMGS*, and *AtHMGR* were downregulated, while those of *AtHDR*, *AtTPS*, and *AtMK* were significantly upregulated (Figure 8). These findings suggest that the overexpression of *PmHDS* induces complex physiological effects on terpenoid synthesis pathways in *A. thaliana*.

## 3. Discussion

Terpenoids, a diverse class of compounds derived primarily from higher plants, have been extensively studied due to their numerous biological activities and applications. To date, tens of thousands of terpenoids have been identified, with many serving as primary metabolites essential for cellular functions in plants [31]. These terpenoids include isoprenoid chains found in quinones (such as ubiquinone and plastoquinone) that are crucial for respiration, photosynthetic pigments like chlorophyll, sterols that predominantly contribute to membrane stability, and hormones that regulate plant growth and development (such as CTK, GA, and ABA) [32]. Additionally, terpenoids play a significant role in various responses to both biotic and abiotic stresses [1,33]. The MEP pathway is a crucial metabolic route for terpenoids’ synthesis across most plants and constitutes an integral aspect of terpenoid research. The *PmHDS* gene, as a key regulator in the MEP pathway, plays a pivotal role in the synthesis of terpenoids. Our study investigated the *PmHDS* gene within the MEP pathway associated with terpenoids’ synthesis in *P. massoniana*, aiming to provide novel insights into terpenoids’ biosynthesis and molecular breeding. In this study, we successfully cloned the *PmHDS* gene and performed comprehensive bioinformatics analyses to gain insights into its structure and function. The conserved domain analysis revealed that the HDS protein is a member of the 4-hydroxy-3-methylbut-2-en-1-yl diphosphate synthase (PRK00694) superfamily, which is known for its role in terpenoid biosynthesis. Multiple sequence alignment results confirmed that the amino acid sequence of the HDS protein is highly conserved and contains several cysteine residues, which are likely to serve as potential binding sites for metal ion transport [29,30].

The expression levels of the *HDS* gene have been shown to vary depending on both plant species and developmental stages. For instance, the *LiHDS* gene in ’Siberia’ lilies exhibits differential expression across various tissues and is influenced by flowering time [20]. In our study, the tissue-specific expression analysis of the *PmHDS* gene in *P. massoniana* revealed that its expression levels in roots and stems were significantly higher than those observed in leaves, which aligns with the expression pattern of *DoHDS* in *Dendrobium officinale* [21], but contrasts with that of *Rauvolfia verticillata* and *Camellia sinensis* [18,19]. This finding suggests that *HDS* exhibits species-specific distribution characteristics within plant tissues and organs. Plant hormones exert a crucial role in regulating gene expression. For instance, hormone regulation will enhance the expression of the *FcHDS* gene in *Fritillaria cirrhosa* [34], which is consistent with the Increased expression level of the *PmHDS* gene in *P. massoniana* due to hormone treatment. Meanwhile, the hormone-induced *HDS* gene expression pattern exhibits certain similarities across species. Following SA treatment, the expression level of the *CsHDS* gene in *Camellia sinensis* was significantly upregulated at 12 h post-treatment and reached its maximum value [19], which was similar to the expression pattern of the same gene in *P. massoniana*.

To further investigate the expression pattern of *PmHDS*, we performed GUS histochemical staining, which revealed that *PmHDS* promoter activity was predominantly localized to the stem of *A. thaliana*. This observation aligns with quantitative data showing elevated *PmHDS* expression within the xylem of *P. massoniana*. This localization may relate to the stress-induced formation of new traumatic resin ducts (TRDs) within conifer xylem, which are known to secrete terpenoids as a defense mechanism against herbivory [25,26,27]. Plant hormones play critical roles in regulating growth, development, and signal transduction processes. Among them, two key plant hormones, SA and MeJA, are recognized for their significant contributions to defense against biotic and abiotic stresses under adverse conditions [35]. In our study, we observed that treatment with MeJA and SA resulted in the upregulation of *PmHDS* expression within needle tissues of *P. massoniana*, indicating that these hormonal signals play a role in inducing *PmHDS* expression, which in turn may regulate terpenoid biosynthesis. The importance of hormonal regulation in plant secondary metabolism is well documented, and our results add to the growing body of evidence supporting this notion. Given the importance of terpenoids in plant defense, it is plausible that SA and MeJA may regulate *PmHDS* expression and, consequently, terpenoid biosynthesis in *P. massoniana*. Future studies are needed to investigate the potential regulatory roles of SA and MeJA on PmHDS expression and terpenoid production in this species. Our quantitative analysis demonstrated that *PmHDS* gene expression was highest in the wood of *P. massoniana*. However, due to the limited availability of experimental materials and the potential negative impact of repeated destructive sampling on plant health, we selected needle tissue, which is also rich in terpenoids and provides sufficient sample numbers, for quantitative analysis in stress treatments. Both resin ducts and needles are active sites for terpenoid synthesis [28], and the *PmHDS* gene is closely associated with terpenoid synthesis, making the choice of needles as the research subject reasonable. Nevertheless, given the high expression level of the gene in the wood, theoretically, using wood as the quantitative analysis material after stress treatment would be more effective. Therefore, future studies will employ more advanced techniques, such as single-cell RNA sequencing, to further investigate the expression pattern of the *PmHDS* gene in wood under stress conditions, thereby enriching and refining our experimental results.

It is common knowledge that the HDS enzyme is a crucial catalyst in the synthesis of the terpenoid precursor IPP and plays a significant role in the production of various secondary metabolites within the MEP pathway, including photosynthetic pigments and hormones. Our study revealed that alterations in *PmHDS* expression levels can significantly impact the production of terpenoid compounds. Specifically, the overexpression of *PmHDS* in transgenic plants led to increased levels of chlorophyll and other terpenoid-derived hormones, such as ABA, SA, and MeJA. These findings are in accordance with those previously reported, where the overexpression of *HDS* in *Dendrobium officinale* enhanced terpenoids’ synthesis through the accumulation of terpene synthase (TPS), and silencing the *LiHDS* gene in ‘Siberia’ lilies led to a significant decrease in the release of terpene-related compounds [20,21]. Collectively, these findings showed the importance of *PmHDS* in terpenoid biosynthesis and plant stress tolerance, highlighting the potential of manipulating *PmHDS* expression to improve plant traits.

Moreover, the results of this study have broader implications for understanding the regulation of terpenoid biosynthesis in plants. By studying the function of *PmHDS* and its role in the MEP pathway of terpenoids’ synthesis, a significant upregulation in the expression levels of HDR, the rate-limiting enzyme located downstream of HDS in the MEP pathway, was observed. HDR directly contributes to the synthesis of terpenoid precursors IPP and DMAPP, and its upregulation suggests that the increased flux through the MEP pathway may be due to the enhanced activity of both HDS and HDR. Concurrently, TCP, a key terpenoid synthase, was also upregulated in the overexpressing plants. Research indicates that TCP is integral to plant growth and responses to environmental stressors [36]. The upregulation of TCP in our study suggests that overexpression of the *PmHDS* gene may also affect plant growth and stress responses through the modulation of terpenoid biosynthesis. Our findings in *P. massoniana* further corroborate the hypothesis that the overexpression of key genes in the terpenoid synthesis pathway can enhance plant resilience against adverse conditions. Specifically, studies have demonstrated that *PmTPS4* and *PmTPS21* play positive regulatory roles in defense against *Bursaphelenchus xylophilus* [37]. This supports our observation that the overexpression of *PmHDS* enhances downstream key gene expression, promotes plant terpenoid synthesis, and bolsters resilience against adverse conditions. However, it is noteworthy that expression levels for *DXR*, an upstream gene in the MEP pathway, were downregulated in our study. This observation implies potential feedback regulation mechanisms within this pathway. Such feedback regulation is common in metabolic pathways to maintain homeostasis and prevent the accumulation of excessive intermediates. Therefore, the downregulation of DXR may be a compensatory response to the increased flux through the MEP pathway due to *PmHDS* overexpression.

Furthermore, while the expression levels of *MK*, a critical gene in the MVA pathway, increased, its upstream counterparts, *HMGS* and *HMGR,* showed decreased expressions. This suggests that the influence of the *HDS* gene on the terpenoid synthesis pathway is indeed a complex regulatory process. The interplay between the MEP and MVA pathways in terpenoid biosynthesis is intricate, and further research is needed to fully understand the mechanisms underlying this cross-pathway regulation. The *PmHDS* gene promoter contains many important cis-regulatory elements that control gene-specific expression, including responses to non-biological stress and plant hormones. In our simulation of adverse stress conditions, *PmHDS* overexpression seedlings were more responsive to stress-induced germination, growth, and development than the wild-type. This further supports the role of PmHDS in enhancing plant resilience and adapting to stressful environments.

In summary, our research findings demonstrate that *PmHDS* can affect the expression of key genes in the terpenoid synthesis pathway downstream, thereby affecting the yield of MEP pathway secondary metabolites and ultimately affecting plant growth, development, and the ability to respond to adverse stress. The complexity of the secondary metabolism pathway and gene regulatory network in *P. massoniana* is further highlighted by these findings. Our study provides a theoretical basis and effective approaches for using molecular breeding technology to cultivate high-yielding and high-resistance *P. massoniana* in the future. By manipulating key genes in the terpenoid synthesis pathway, it may be possible to develop pine varieties with enhanced resilience to pests, diseases, and environmental stressors. Such advancements could have significant implications for forestry and agriculture, contributing to sustainable forest management and improved crop productivity.

## 4. Materials and Methods

### 4.1. Plant Materials

We acquired two-year-old pine seedlings from Baisha National Forest Park (latitude: 25°08′58″, longitude: 116°35′24″, Shanghang City, China) and sowed them in pots filled with black carbon soil, perlite, and vermiculite in a ratio of 4:2:1. The environmental parameters were adjusted to a relative humidity ranging from 60% to 70%, a temperature of 25 ± 2 °C, and a light regime of 16 h of light and 8 h of darkness. Once the pine seedlings had grown for approximately one month and reached a stable state, RNA and gDNA were extracted from the needles for the cloning of the open reading frame (ORF) and promoter of *PmHDS*. Furthermore, tissue samples were gathered from 15-year-old pine trees cultivated in the nursery of Nanjing Forestry University (latitude: 32°47′12″ N; longitude: 118°49′ E) for tissue-specific real-time quantitative PCR analysis.

For heterologous expression studies, *A. thaliana* of the Columbia (Col-0) genetic background was used. The overexpression vector PBI121 was transformed into Agrobacterium GV3101 using the flower dip method [38]. Transgenic plants were selected from the 1/2MS medium containing 40 μg/mL kanamycin. Two weeks later, transgenic plants confirmed by genomic PCR were transplanted into pots for continued growth until T2-generation seeds were harvested for subsequent experiments.

### 4.2. Cloning of PmHDS and Its Promoter

RNA was extracted from the tissues of 2-year-old *P. massoniana* seedlings using the FastPure Universal Plant Total RNA Isolation Kit (Vazmy Biotechnology, Nanjing, China). cDNA synthesis was performed on total RNA through reverse transcription with the One-step gDNA Removal and cDNA Synthesis SuperMix Kit (Yeasen Biotechnology, Shanghai, China). Specific primers were designed utilizing Primer Premier 5.0 software (Table S2). The open reading frame (ORF) sequence of *PmHDS* was cloned from *P. massoniana* using a 50 μL PCR system based on the synthesized cDNA. The PCR conditions were as follows: pre-denaturation at 94 °C for 30 s, followed by 35 cycles consisting of denaturation at 98 °C for 10 s, annealing at 58 °C for 15 s, extension at 72 °C for 40 s, and a final extension at 72 °C for an additional five minutes. The amplified ORF fragment was ligated into the Blunt vector using pCE3-blunt (Vazmy Biotechnology, Nanjing, China).

To clone the promoter region of *PmHDS,* the gene fragment with the highest similarity upstream of approximately 2000 bp was identified by using the *P. tabuliformis* genome and used as a reference sequence for designing specific primers. Genomic DNA (gDNA) was extracted from *P. massoniana* leaves serving as the PCR template. The PCR reaction products were dispatched to Tsingke Biotechnology, Nanjing, China, for sequencing. The sequencing outcomes were compared against our known sequences, and the sequences that were correctly matched were assembled jointly using the online software SeqMan 1.0.

### 4.3. Bioinformatics Analysis of the PmHDS Protein Sequence

The physical and chemical properties of the PmHDS protein were analyzed through the Expasy online platform (https://www.expasy.org, accessed on 4 July 2024). Hydrophobicity analysis was carried out using ProtScale (https://web.expasy.org/protscale/, accessed on 8 July 2024). Signal peptides were identified by SignalP4.1 (https://services.healthtech.dtu.dk/services/SignalP-4.1/, accessed on 8 July 2024), while transmembrane domains were analyzed through TMHMH-2.0 (https://services.healthtech.dtu.dk/services/TMHMM-2.0/, accessed on 5 October 2024). The secondary structure of the protein was evaluated by SOPMA (https://npsa-prabi.ibcp.fr/cgi-bin/npsa_automat.pl?page=npsa%20_sopma.html, accessed on 8 July 2024). Tertiary structure models for PmHDS were constructed with SWISS-MODEL (https://swissmodel.expasy.org/, accessed on 5 October 2024). The nucleotide sequence of PmHDS was compared to other sequences utilizing the Blastn tool from NCBI (https://www.ncbi.nlm.nih.gov/, accessed on 29 June 2024). Amino acid sequences underwent multiple sequence alignment with ClustalX2.1 and ESPript 3.0 online tools (https://espript.ibcp.fr/ESPript/index.php/, accessed on 5 October 2024). MEGA11.0 software was used to construct an evolutionary tree based on the *PmHDS*-encoded amino acid sequences, and a phylogenetic analysis was performed using the neighbor-joining method. The bootstrap method was applied for evaluation, with a test count set to 1000; gaps were treated with complete deletion. The evolutionary tree was visualized using the online drawing software iTOL v7 (https://itol.embl.de, accessed on 9 October 2024). Additionally, protein–protein interactions (PPIs) were predicted and analyzed using the STRING database (https://cn.stringdb.org/cgi/input?sessionId=bop583S4w1aI&input_page_active_form=multiple_sequences, accessed on 29 June 2020) and the Cytoscape software (version Cytoscape_v3.8.2) [39,40].

### 4.4. Non-Biological Stress and Hormone Treatment Experimental Materials

Healthy two-year-old *P. massoniana* seedlings were selected for six treatments, each consisting of three seedlings, conducted in a culture room at 25 °C under a 16 h/8 h light-dark cycle. Despite our quantitative analysis indicating that *PmHDS* gene expression was highest in the xylem (Figure 3b), given the limited availability of xylem samples from two-year-old seedlings, we were concerned that repeated destructive sampling might compromise the integrity of our experimental results. Therefore, to minimize the impact on the xylem and ensure the reliability of the data, we selected needles with similar terpenoids and a sufficient sample size for stress quantitative analysis. The first treatment involved drought stress, with leaf samples collected on the 3rd, 7th, 12th, and 20th days after watering ceased. The second treatment induced osmotic stress by immersing the seedlings in a 150 mM NaCl solution. Treatments three to six involved spraying the needles with 100 μM of ABA, 50 μM of ETH, 10 mM of MeJA, and 1 mM of SA [41]. Samples from treatments two to six were collected at intervals of 0 h, 3 h, 6 h, 12 h, and 24 h post-treatment. All samples were immediately flash-frozen in liquid nitrogen and stored at −80 °C for later experiments.

### 4.5. Real-Time Quantitative PCR Analysis

Specific primers, q*PmHDS*-F and q*PmHDS*-R (Table S2), were designed using Primer Premier 5.0 software for qRT-PCR. The target gene expression was detected using the StepOnePlus TM real-time PCR system (Applied Biosystems Inc., Foster, CA, USA) equipped with a laptop. The Hieff UNICON^®^ Universal Blue qPCR SYBR Green Master Mix (Yeasen Biotech, Shanghai, China) was used in the qPCR reaction. *PmTUA* gene was used as an internal reference gene (NCBI accession number: KM496535.1) (Table S2), and *AtActin2* was used for the qPCR analysis of the *PmHDS* gene in *A. thaliana*. The PCR reaction consisted of a total volume of 10 μL, including 1 μL of 20-fold diluted cDNA, 5 μL of SYBR Green real-time PCR master mix, 0.4 μL of each 10 μM primer, and 3.2 μL of ddH_2_O. The reaction system and amplification protocol were followed according to the instructions in the manual of Hieff UNICON Universal Blue qPCR SYBR Green Master Mix (Yeasen Biotech, Shanghai, China).

Relative transcript abundance was quantified using the 2^−ΔΔCT^ method. Each qRT-PCR result was derived from three biological replicates and three technical replicates. The biological replicates consisted of samples of *P. massoniana* collected from different individuals under identical growth conditions and tissue types. The technical replicates served as experimental repetitions. Data analysis was performed using Excel 2021, while figures were generated in GraphPad Prism 8.0.2.

### 4.6. Determination of Photosynthetic Pigment Content and Terpenoid Synthesis Hormone Content

To determine the photosynthetic pigment and terpenoid hormone contents, leaf samples were collected from transgenic and wild-type plants. Seeds of transgenic A. thaliana and Col-0 were surface-sterilized by soaking in 5% sodium hypochlorite for 8 min, followed by treatment with 75% alcohol for 10 min. The sterilized seeds were sown on solid 1/2MS medium (1/2MS salts, 1% sucrose, 0.6% agar, pH 5.8) [42]. The plates were vernalized in the dark at 4 °C for 3 days to synchronize germination and then transferred to a light incubator (16 h light/8 h darkness) at 24 °C for one week. Subsequently, the seedlings were transplanted into soil for further growth. Photosynthetic pigments (chlorophyll a, chlorophyll b, and carotenoids) were extracted and quantified spectrophotometrically using standard methods. Additionally, terpenoids’ hormone contents were analyzed using appropriate extraction and quantification techniques, such as gas chromatography–mass spectrometry (GC-MS) or liquid chromatography–mass spectrometry (LC-MS), to assess the impact of *PmHDS* overexpression on terpenoids’ synthesis in the transgenic *A. thaliana* plants.

The method for measuring photosynthetic pigments follows Gan [43], with slight modifications. Take 0.1 g of leaves and place them in a 2.0 mL centrifuge tube containing two 3mm grinding steel balls (Yeasen Biotech, Shanghai, China). Quickly freeze the tube in liquid nitrogen and grind the tissue into powder using a high-throughput grinder. Gradually add 95% ethanol until the powder is completely dissolved; then, adjust the volume to 10 mL with ethanol and shake gently for 10 min. Invert the tube and let it stand for another 10 min until it turns white; keep this process in darkness. Transfer the extract to a cuvette with a light path of 1 cm; this serves as the experimental group while using pure ethanol as a control group. Measure absorbance at wavelengths of 665 nm, 649 nm, and 470 nm using spectrophotometry. The formula for calculating photosynthetic pigments is based on a reference.

### 4.7. GUS Staining

According to the description in Section 4.6, the constructed GUS-tagged overexpression vector was transformed into Agrobacterium GV3101. The wild-type *A. thaliana* plants were subjected to genetic transformation using the floral bud infection method [41]. Following screening, transgenic plants were identified and confirmed through genomic PCR analysis. These confirmed transgenic plants were subsequently transplanted into pots for continued growth. After harvesting the T2 generation, *A. thaliana* was immersed in a GUS staining solution (Leagene Biotechnology, Beijing, China) and incubated overnight at 37 °C in darkness. Subsequently, the plants underwent treatment with a bleaching solution composed of a V(ethanol)/V (acetic acid) ratio of 3:1. Upon completion of bleaching, photographs were taken and recorded using a microscope.

### 4.8. Relative Expression Levels of Genes Related to Terpenoid Synthesis Pathways

The relative expression levels of genes related to terpene synthesis pathways were determined by the qRT-PCR method. The *A. thaliana* genes selected included *AtDXR* and *AtHDR* in the MEP pathway, *AtHMGS*, *AtHMGR*, and *AtMK* in the MVA pathway, *AtGGPPS* in the GGPP pathway, and *AtTPS* and *AtPSY* in the lycopene pathway (Table S3. The method and reaction system used for qRT-PCR are consistent with those described in Section 4.5.

### 4.9. Statistical Analysis

Each experimental result was obtained from three biological replicates and three technical replicates. The data were processed using Excel and graphed using GraphPad Prism 8.0.2. “*” indicates the *p*-value of the statistical test, where * *p* < 0.05, ** *p* < 0.01, and *** *p* < 0.001.

## 5. Conclusions

This study elucidates the pivotal role of HDS as a key enzyme in the MEP pathway for terpenoid synthesis in *P. massoniana*. The PmHDS protein domain is highly conserved and contains multiple cysteine residues potentially serving as binding sites for metal ions. Based on quantitative tissue expression analysis, we proposed that *PmHDS* exhibits temporal and spatial expression patterns in the xylem, stem, leaf, and root of *P. massoniana*, which was subsequently validated through GUS staining of its promoter. The overexpression of *PmHDS* in *A. thaliana* demonstrated heightened sensitivity to drought and stress conditions induced by SA and MeJA. Notably, we observed an upregulation of downstream key genes associated with the MEP pathway for terpenoids’ synthesis in transgenic *A. thaliana*. Furthermore, we have observed that transgenic plants appeared more verdant and exhibited superior growth compared to wild-type plants, which might be associated with the significant enhancement of HDS enzyme activity and the elevated levels of photosynthetic pigments and related terpenoid derivatives. These findings collectively emphasize the critical role of *PmHDS* in regulating metabolic processes involved in terpenoid synthesis and stress responses. In conclusion, this research contributes to a deeper understanding of the regulatory mechanisms governing terpenoid biosynthesis pathways in *P. massoniana*, providing valuable insights for future molecular breeding efforts aimed at developing high-yielding and disease-resistant varieties.

## Data Availability

Data are contained within the article or Supplementary Material.

## Supplementary Materials

The following supporting information can be downloaded at: https://www.mdpi.com/article/10.3390/ijms26020457/s1.
