# Supplementary material for: Characteristics and Functions of PmHDS, a Terpenoid Synthesis-Related Gene in Pinus massoniana Lamb
Source: Int J Mol Sci. 2025 Jan 8;26(2):457. doi: 10.3390/ijms26020457 (PMC11764807; doi:10.3390/ijms26020457)
Supplement: Supplementary file 1 [file ijms-26-00457-s001.zip › Supplementary figures.pdf]

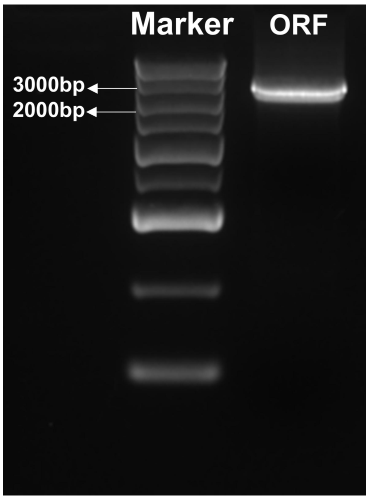

Figure S1. Cloning of the open reading frame of *PmHDS*. Marker: DNA Marker (100~5000 bp) ; ORF: open reading frame for *PmHDS*.

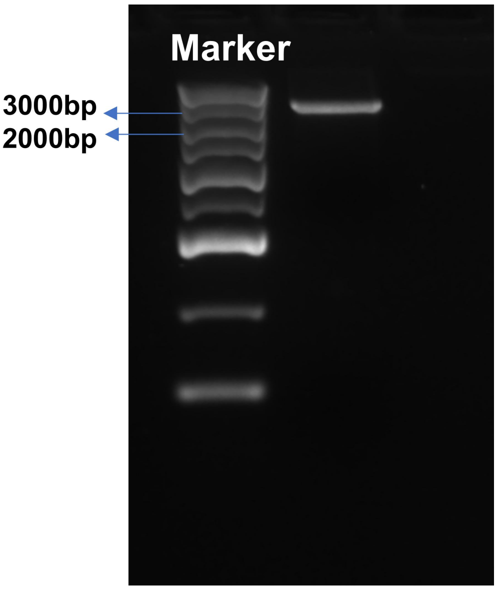

Figure S2. Cloning of the *PmHDS* promoter

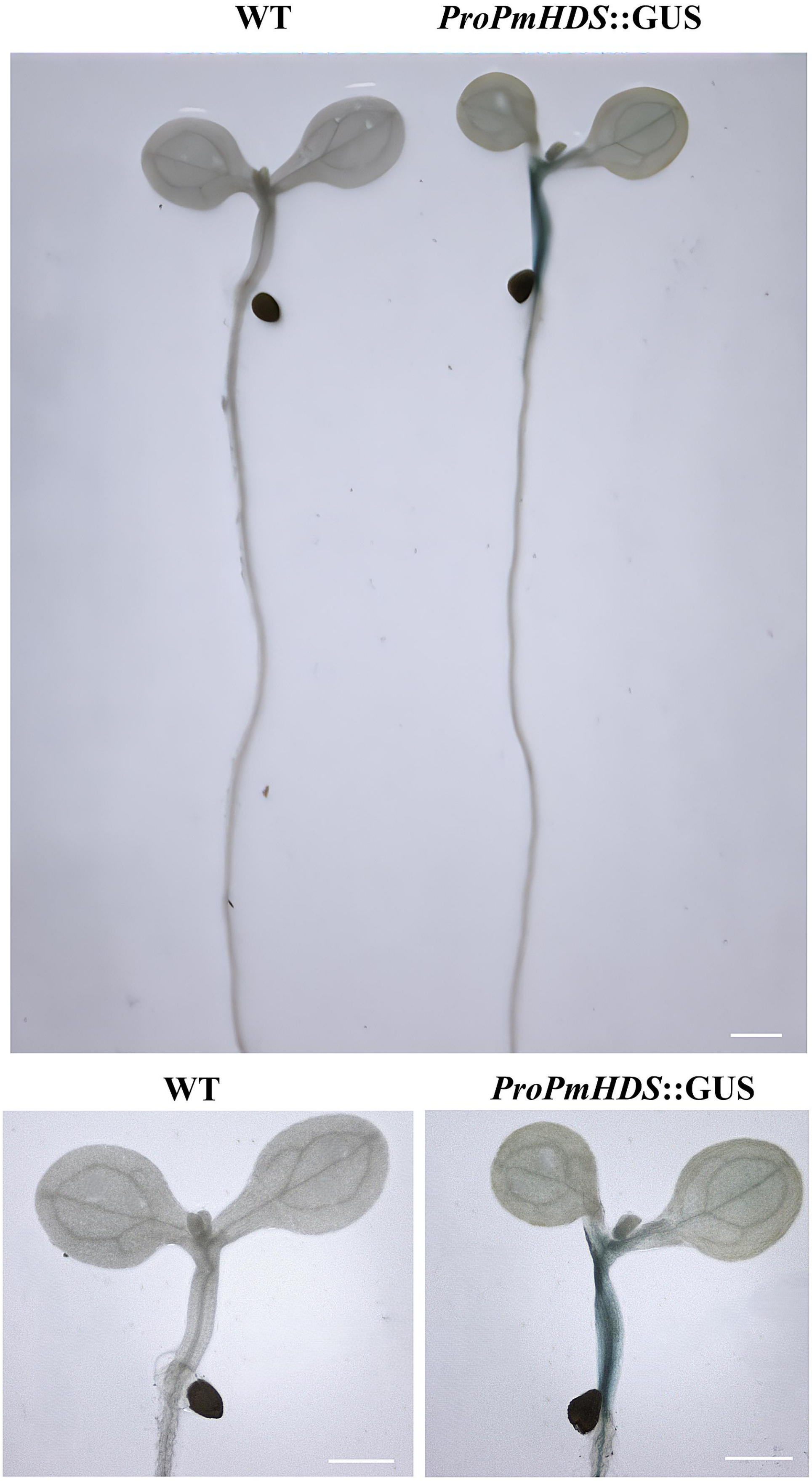

Figure S3. Analysis of pBI121-*proPmHDS*::GUS Staining, scale bar = 2 mm.

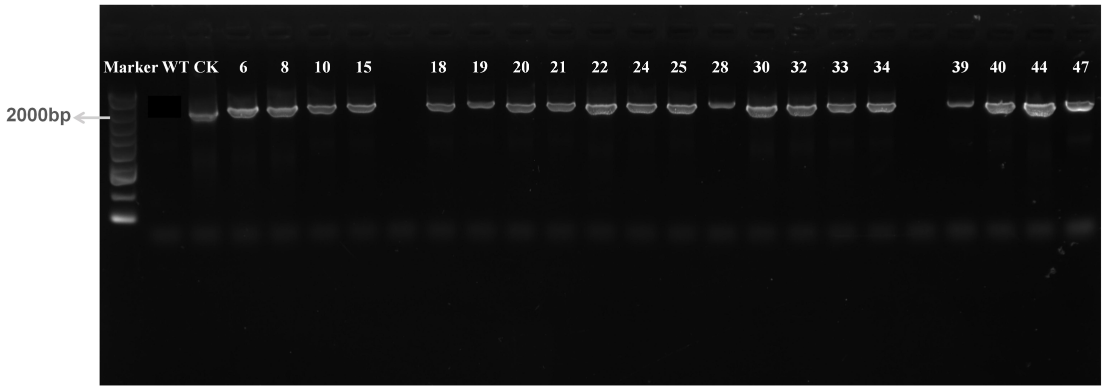

Figure S4. PCR assay for overexpression of *PmHDS Arabidopsis thaliana*. CK: positive control; WT: wild type (negative control); 6 、 8 、 10 、 15 、 18~22 、24 、25 、28 、30 、32~34 、39 、40、 44 、47: transgenic strain.

>*PmHDS*

ATGGCTGCTGGAACAATTCCTGCTTCGTTCTCTGGTTTGAAGAAAACCGATTCCTTGCC
AGGCTGCTTGCAGAGTACCCATGCTAGAAAAGGCTTTGCTATGCATAACTTTCAGAATC
CGAGGTCATCGTCATTCCGGATAAGGTTTAGGGGGGTCAGGAACTCATCCTCAGACATT
GCAGAAATGAAGCCAGCTTCTGAGGGCAGTCCACTTTTGGTTCCCAGGCAGAAGTATT
GTGAATCCATATACAAGACAATACGCAGAAAAACACGTACAGTGATGGTTGGCAATGT
CCCACTTGGGAGCGAACATCCTATCAGGGTTCAAACAATGACTACTACTGATACAAAG
GATGTTCTTGGTACCGTAGAACAGGTCATGCGAATAGCCGATAAAGGAGCAGATCTTGT
TCGAATCACTGTTCAGGGAAAAAAAGAAGCAGATGCTTGCCATGACATTAAAAATACC
CTTGTACAGAAGGGCTATGATATTCCACTTGTGGCAGACATCCATTTTGCTCCTTCAGTT

GCATTGCGTGTTGCAGAATATTTTGATAAGATCCGCGTTAATCCTGGAAATTTTGCTGAC
AGGCGTGCCCAATTTCAGAAACTAGAATACACAGAGGAAGACTATCAGAAGGAGCTT
GAACACATTGAAGAGGTTTTCACACCATTGGTGGAGAAATGCAAGAAGTACGGAAGG
GCAATGCGAATTGGCACAAACCATGGAAGTCTTTCCGATCGTACTATGAGTTACTATGG
TGATTCTCCCAGGGGTATGGTGGAATCAGCATTTGAATTTGCACGCATTTGCCGGAAGT
TGGATTTTCATAATTTTGTGTTTTCAATGAAAGCGAGCAATCCTGTAGTCATGGTTCAGG
CATACCGTTTACTTGTTGCGGAGATGTATGTCCAAGGATGGGATTATCCATTGCATTTAG
GAGTTACTGAAGCTGGTGAAGGTGAAGATGGACGCATGAAGTCTGCAATTGGCATTGG
AACACTTTTGCAGGATGGTTTGGGTGATACTATTCGAGTTTCCCTTACAGAACCTCCAG
AAGAGGAGATTAATCCCTGTAGAAGACTTGCAAATCTTGGGATGCAAGCTGCAAAGCT
AGGGAAAGGAGTGGCTCCTTTTGAGGAGAAGCATCGTCATTACTTTACTTTCCAACGC
AGGACTGGCCAGCTTCCAGTACAGAAGGAGGGTGATGAGGTGGATTACAGAGGAGTT
CTGCATCGTGATGGTTCTGTTCTAATGTCAGTGTCCCTTAACCAGTTGAAGACACCAGA
GATTTTGTACAAATCACTGGCAACGAAGCTTATTGTCGGCATGCCCTATAAGGATCTTG
CAACAGTGGACACAATATTGTTGAGGGAACTTCCCCCGGCAGAGGACACCGAAGCGA
GGCTGGCGATTAAAAGGCTTATAGACATAAGTATGGGCGTTTTGACACCTTTATCAGAG
CAACTTACCAAACCTTTGCCAAATGCCATGGTCCTTGTAAATTTGAAGGAACTTTCAAC
TGGTGCTCACAAGCTTCTACCAGCAGGTACACGTTTGGCTGTAACTCTTCGAGGAGAT
GAAACATATGAGGAATTGGATGTCATCAAGAGTGTGGATGCCACAATGATTCTCTTAAA
TCTGCCTAGAGAAGAAGAGAAAGTTAACCGTGTCCATGCATCTCGAAGATTGTTTGAG
TACTTGCAAGAAAATGCACTCAACTTCCCCGTTATTCATCACATACAGTTTCCTGAAGG
AACTCACAGGGATGATCTAGTCATCAGTGCAGGCAGTGAGGCAGGTGCTCTCCTTGTC
GATGGTCTTGGTGATGGCCTAATGTTGGAGGCTTCAGACCAAGACTTCGATTTTCTTCG
CAACACATCGTTTGGACTGCTCCAAGGATGCAGGATGCGCAATACAAAAACGGAGTAT
GTATCATGCCCATCATGTGGCCGCACTCTGTTTGACCTCCAAGAAATAAGTGCAGAGAT
TCGGGCGAAAACACAGCATCTTCCTGGTGTTTCGATTGCTATCATGGGCTGCATTGTGA
ATGGTCCTGGAGAGATGGCAGATGCAGACTTTGGATATGTTGGTGGGTCACCAGGAAA
GATTGATCTCTATGTTGGAAAGGAAGTTGTCAAAAGAGGTATAGCAATGGAGCAAGCA
TGTGATGCTTTAATTCAGTTGATTAAGGACTATGGGCGATGGGTCGATCCTCCTGCTGA
AGAATAA

>*Pro-PmHDS*

CAGCCTGGCAAGGAATCGGTTTTCTTCAAACCAGAGAACGAAGCAGGAATTGTTCCA
GCAGCCATCTTGTCAACTATCCACTTCAAATCAGAGGTCCAAATGAAACGAAAACAGC
TTTGCAGAGTGAATCCCTGTTGCTTTTAGCTGCTATTGATAGCTTTACAGTTGAGTTGCT
GCAGAAATTCTGCAGAAGAAATGAAATGGATGTTGCAGGCGTGGCTGCAAGCAATGA
GATGAGACTGGATGAAATCCAGTCATTTATTGAGAATTAGCATGGGGTATAGTCAGATA
AGACAAGACGAAGGATCTAGGGTGTATATACCACTACCGAATTACAAACTTGGAGTCG
TTTATTTGTGGGTATAATTTGCATTTTGTAGAGAATCTTTGGCAGAGCAGGTCAGGACA
AACCCAACTCCCCTGCAATTTTCCACAGACAAGTTTTTCATCCGATCCTACCATTGTGA
AATGGACAAATTAAGGGCATATTCCCATGTACATTTCATATTGTCACGAGCACATAAAAT
AATTTGTGGGAAATTTTGGTGCGTTGACATGGATAATCTGTTATATTGATTGCGATGTTC
ACCCATGTAATCACATGGGAGGATTTCAGTTTTCATTTTCTCTTTTAATGTAGGCCATGC
TTAGGGTTGAGGGCAAATTGCCCTTTTCTATGAGGTTCAGTAAAAACTTTCATGGGTTA

ACCAGATCAGATTCTTAACAAGTATTCAATTATTTTAGTTATAGGGAAATTAAGATGTTTT
ATAATTTAATATTTTATAGGTTTAGAAATGAGATTTTTGTGATTTATATGATAGATAAATGT
CTTTCCGATTAATCACATTATTAAGTTTTGGTCTACGTTTCGAATCAACTAATATGGTTCA
TCCTCAGACCTTTGTCCTCAGGATGAACCACATTAATTGGTTCGAAACGTGGACCAAAT
CCTAATAGTGTGAATAATCTGAAAGATATTTATTTTATATTTTATATTAAAATAATAAAATA
AAACTCATTAAAAAATATTAATAAAAAACTTTTAATATTTATTATAAAATTTAAATTTTTA
ATATATGTCTATTTTAAACAACATAATTTTACATCAAATTTTTATATAGAGAAAGATAAAA
TAATTGAAATTAAGAATTTATAATTCCATCCACATTCACTTAATATTTTTTATCATAAAAA
AAATATAAAAAATATCATAAAAAAATATAAAAAATATGTGTAATTCGTCCCCAAATAAAA
TTAACATATGCTATTTAAAAAAATAAATGTGTTGATCAAAATATATTTATGTGTATTAATA
CTCTTTATATAAAAAGCAAAATAGATTTATATTTTATTAAGAGGAATTTGCCTTTCTCTAT
GTTATTAGATTCCTTAAACTTAATTAGATTCCTTAAACTTAATTAGTGATCCATTATTTAA
CTTATTAGGTTTAATAATCTTTTATTTTATATATATATGCTTATTATTATATCAATTATTTATGT
TTTTATTTGTTTAGTTTTAATAGTAGATAAGTTTATTAATAGGTTTTGGGAGTTAATTCTTA
CTATTTATTTTATTATATAACAAGAAACTAATTTTTTTTTCACCTAGGTGGAAAAACCTAT
CAACTCAGGTGTCAAAACCAACAGTGAGGACAGCCATCTCTGGCTGCTATCCGCGAAT
GTCGGTTTGACTTACATAATAACTAACTACCTAATTATTTATATATTTATATTTAATTCTGA
ATATTTATATTTATATATTATTCCATATTATATTATTTATATATTTATATTTAATTCTGAATATTT
ATATTTATATATTATTCCATATTATATTATTGATTTAAGGTTTTTGATCTTACAATTATATATAT
AATTAAATTCATAATACACATACAATTATTTATTATTCCATATTATGATTTCAATTATCAAA
GAAAAAGTTTTATATGGTTTTTTCATATTAGATTAATAACTAATGTTTGTTTTATTGGTTTT
GGTAGTTATTTCAATCAATTATATTTTTTGGGAAATAAATATAACAAAAACTAGCTTACTT
ATTAATTAACTAACCATTTATTTATATAATTATAATTATGGAAATAGGCCTATTAATAACTA
ATTGATTCATATTCTTGATTATTAAATTCAAACTTTACCTTTATTCAAACTTTGGTAATAAA
TATAACAAAATTTTGGTAATAATTATAACAAA
